# A note on the stationary distribution of stochastic SEIR epidemic model with saturated incidence rate

**DOI:** 10.1038/s41598-017-03858-8

**Published:** 2017-06-21

**Authors:** Qixing Han, Liang Chen, Daqing Jiang

**Affiliations:** 1 School of Mathematics, Changchun Normal University, Changchun, 130032 China; 2 School of Mathematics and Statistics, Northeast Normal University, Changchun, 130024 China; 3 College of Science, China University of Petroleum(East China), Qingdao, 266580 China; 4 Nonlinear Analysis and Applied Mathematics (NAAM)-Research Group, King Abdulaziz University, Jeddah, Saudi Arabia

## Abstract

The stochastic SEIR infectious diseases model with saturated incidence rate is studied in this paper. By constructing appropriate Lyapunov functions, we show that there is a stationary distribution for the system and the ergodicity holds provided ${R}_{0}^{s}$ > 1. In particular, we improve the results obtained by previous studies greatly, condition in our Theorem is more concise and elegant.

## Introduction

The study of the epidemic models have long been and will continue to be one of the dominant themes in mathematical biology due to its importance at both understanding the spread and control of infectious diseases in a community. Many researchers have made a significant progress on *SIR* models^1–6^, where *S*, *I*, *R* denote the fractions of the susceptibles, the infectives and the recovered hosts in the population respectively. *SIR* models assume the disease has no latent period. However, for some diseases, such as hepatitis B, AIDS, sometimes has to be passed before an infected individual becomes infectious. Therefore, an extra class, the class of exposed hosts (*E*), should be added to the system, where *E* denotes the fraction of the exposed population. The model is called *SEIR* (susceptible-exposed-infected-removed) model, and *SEIR* models were investigated by many researchers^7–9^.

On the other hand, the incidence rate palys an important role in the epidemics models. some authors employ the bilinear incidence rate *βSI* ^10, 11^. After studying the cholera epidemic spread in Bari in 1973, Capasso and Serio^12^ introduced a saturated incidence rate *g*(*I*)*S* into epidemic models, where *g*(*I*) tends to a saturation level when *I* gets large, i.e.

$$g(I)S=\frac{\beta IS}{1+\alpha I}$$

where *βI* measures the infection force of the disease and $\tfrac{1}{1+\alpha I}$ measures the inhibition effect from the behavioral change of the susceptible individuals when their number increases or from the crowding effect of the infective individuals. Then the *SEIR* model with a saturated incidence rate can be described as follows:

11 $$\{\begin{array}{ccc}dS & = & (\lambda -\frac{\beta SI}{1+\alpha I}-{d}_{S}S)dt,\\ dE & = & [\frac{\beta SI}{1+\alpha I}-({d}_{E}+\theta )E]dt,\\ dI & = & [\theta E-({d}_{I}+\delta +\gamma )I]dt,\\ dR & = & (\gamma I-{d}_{R}R)dt.\end{array}$$

The parameters in the model are positive constants and summarized in the following:

$$\begin{array}{l}\quad \quad \quad \quad \,\,\lambda :{\rm{the}}\,{\rm{birth}}\,{\rm{rate}}\,,\\ {d}_{S},{d}_{E},{d}_{I},{d}_{R}:{\rm{natural}}\,{\rm{death}}\,{\rm{rates}}\,{\rm{of}}\,S,E,I,R,{\rm{respectively}},\\ \quad \quad \quad \quad \,\,\delta :{\rm{additional}}\,{\rm{disease}}\,{\rm{caused}}\,{\rm{rate}}\,{\rm{suffered}}\,{\rm{by}}\,{\rm{the}}\,{\rm{infectious}}\,{\rm{individuals}},\\ \quad \quad \quad \quad \,\,\theta :{\rm{the}}\,{\rm{rate}}\,{\rm{at}}\,{\rm{which}}\,{\rm{the}}\,{\rm{exposed}}\,{\rm{individuals}}\,{\rm{become}}\,{\rm{infectious}},\\ \quad \quad \quad \quad \,\,\gamma :{\rm{the}}\,{\rm{recovery}}\,{\rm{rate}}\,{\rm{of}}\,{\rm{infective}}\,{\rm{individuals}}{\rm{.}}\end{array}$$

Define the basic reproductive number ${R}_{0}=\frac{\lambda \beta \theta }{{d}_{S}(\theta +{d}_{E})({d}_{1}+\delta +\gamma )}$. The dynamical behavior of model (1.1) is as follows ref. 13:

If *R*_0_ < 1, system (1.1) has a unique disease-free equilibrium ${P}_{0}=(\frac{\lambda }{{d}_{S}},0,0,0)$, which is a global attractor in the first octant.

If *R*_0_ > 1, then model (1.1) has two equilibria, a disease-free equilibrium *P*_0_ and an endemic equilibrium P* = {S*, E*, I*, R*}. *P*_0_ is unstable and P* is a global attractor in the interior of the first octant.

Some authors take stochastic perturbation into account when they investigate the epidemics system^14–21^. In this paper, we assume that the perturbation is of white noise type, that is, ${d}_{S}\to {d}_{S}+{\sigma }_{1}{\dot{B}}_{1}(t)$, ${d}_{E}\to {d}_{E}+{\sigma }_{2}{\dot{B}}_{2}(t)$, ${d}_{I}\to {d}_{I}+{\sigma }_{3}{\dot{B}}_{3}(t)$, ${d}_{R}\to {d}_{R}+{\sigma }_{1}{\dot{B}}_{4}(t)$, then we get the following stochastic system

12 $$\{\begin{array}{ccc}dS & = & (\lambda -\frac{\beta SI}{1+\alpha I}-{d}_{S}S)dt+{\sigma }_{1}Sd{B}_{1}(t),\\ dE & = & [\frac{\beta SI}{1+\alpha I}-({d}_{E}+\theta )E]dt+{\sigma }_{2}Ed{B}_{2}(t),\\ dI & = & [\theta E-({d}_{I}+\delta +\gamma )I]dt+{\sigma }_{3}Id{B}_{3}(t),\\ dR & = & (\gamma I-{d}_{R}R)dt+{\sigma }_{4}Rd{B}_{4}(t),\end{array}$$

where ${B}_{1}(t)$, ${B}_{2}(t)$, ${B}_{3}(t)$, ${B}_{4}(t)$ are standard one-dimensional independent Wiener processes, ${\sigma }_{i}({\sigma }_{i} > 0),i=1,2,3,4$ are the intensity of the white noise.

In ref. 17, Yang *et al*. show that there is a stationary distribution *μ*(·) for system (1.2) and it has ergodic property provided the following conditions hold:

$$\begin{array}{cc}({\rm{H}}1) & {R}_{0} > 1;\end{array}$$

$$\begin{array}{ll}({\rm{H}}2) & {\rm{\min }}\{{k}_{1}{S}^{\ast 2},{k}_{2}{E}^{\mathrm{\ast 2}},{k}_{3}{I}^{\ast 2},{k}_{4}{R}^{\ast 2}\} > \rho  > 0\end{array},$$

where (S*, E*, I*, R*) is the interior equilibrium of system of (1.1), ${k}_{1}={d}_{S}-{\sigma }_{1}^{2}-\frac{{\sigma }_{1}^{2}{({d}_{E}+{d}_{S}+\theta )}^{2}{S}^{\ast }}{{d}_{S}({d}_{E}+\theta )}$, ${k}_{2}=\frac{{d}_{E}+\theta }{4}-{\sigma }_{2}^{2}$, ${k}_{3}=\frac{({d}_{I}+\delta +\gamma )({d}_{E}+\theta )}{8{\theta }^{2}}({d}_{I}+\delta +\gamma -4{\sigma }_{3}^{2})$, ${k}_{4}=\frac{{d}_{R}{({d}_{I}+\delta +\gamma )}^{2}({d}_{E}+\theta )}{8{\theta }^{2}{\gamma }^{2}}({d}_{R}-2{\sigma }_{4}^{2})$, and

$$\begin{array}{rcl}\rho  & = & {\sigma }_{1}^{2}[\frac{{({d}_{E}+{d}_{S}+\theta )}^{2}{S}^{\ast 3}}{{d}_{S}({d}_{E}+\theta )}+{S}^{\mathrm{\ast 2}}+\frac{\beta {S}^{\mathrm{\ast 2}}{I}^{\ast }}{{d}_{S}\mathrm{(1}+{\alpha }^{\ast })}]\\  &  & +{\sigma }_{2}^{2}[{E}^{\mathrm{\ast 2}}+\frac{{S}^{\ast }{E}^{\ast }}{2}+\frac{\beta {S}^{\ast }{E}^{\ast }{I}^{\ast }}{2{d}_{S}\mathrm{(1}+{\alpha }^{\ast })}]\\  &  & +{\sigma }_{3}^{2}[\frac{({d}_{I}+\delta +\gamma )({d}_{E}+\theta ){I}^{\mathrm{\ast 2}}}{2{\theta }^{2}}\\  &  & +\frac{{S}^{\ast }{I}^{\mathrm{\ast 2}}{({d}_{E}+{d}_{S}+\theta )}^{2}}{4\theta {d}_{S}}(1+\frac{\beta }{{d}_{S}\mathrm{(1}+{\alpha }^{\ast })})]\\  &  & +\frac{{\sigma }_{4}^{2}{d}_{R}{({d}_{I}+\delta +\gamma )}^{2}({d}_{E}+\theta ){R}^{\mathrm{\ast 2}}}{4{\theta }^{2}{\gamma }^{2}}.\end{array}$$

The main aim of this paper is to deal with the existence of stationary distribution of system (1.2). By construct new Lyapunov functions and rectangular set, instead of elliptical region, our results do not depend on the equilibrium P* of the deterministic system (1.1), which will improve the above result to a great extent.

## Ergodic Properties

In order to show the existence of a stationary distribution, firstly, we cite a known result from ref. 22 as a lemma.

Let *X*(*t*) be a homogeneous Markov Process in *E*_*l*_ (*E*_*l*_ denotes *l* dimensional Euclidean space), and is described by the following stochastic equation:

21 $$dX(t)=b(X)dt+\sum _{r=1}^{k}\,{g}_{r}(X)d{B}_{r}(t\mathrm{).}$$

The diffusion matrix is defined as follows:

$${\rm{\Lambda }}(x)=({\lambda }_{ij}(x)),\,{\lambda }_{ij}(x)=\sum _{r=1}^{k}\,{g}_{r}^{i}(x){g}_{r}^{j}(x\mathrm{).}$$

**Lemma 2.1**. *The Markov process X*(*t*) *has a unique ergodic stationary distribution μ*(·) *if there exists a bounded domain U* ∈ *E*_*l*_ *with regular boundary* Γ *and*

*A*_1_: *there is a positive number M such that* $\sum _{i,j=1}^{l}\,{\lambda }_{ij}(x){\xi }_{i}{\xi }_{j}\ge M{|\xi |}^{2},\,x\in U,\,\xi \in {R}^{l},$

*A*_2_: *there exist a nonnegative C* ^2^–*function V such that LV is negative for any E*_*I*_\*U*. *Then*

$${P}_{x}\{\mathop{\mathrm{lim}}\limits_{T\to \infty }\frac{1}{T}{\int }_{0}^{T}\,f(X(t))dt={\int }_{{E}_{l}}\,f(x)\mu (dx)\}=1$$

*for all x* ∈ *E*_*l*_, *where f*(·) *is a function integrable with respect to the measure μ*.

**Theorem 2.1**. *Assume that*

$${R}_{0}^{s}:=\frac{\lambda \beta \theta }{({d}_{S}+\frac{{\sigma }_{1}^{2}}{2})({d}_{E}+\theta +\frac{{\sigma }_{2}^{2}}{2})({d}_{I}+\delta +\gamma +\frac{{\sigma }_{3}^{2}}{2})} > 1,$$

*then for any initial value* $(S\mathrm{(0)},E\mathrm{(0)},I\mathrm{(0)},R\mathrm{(0))}\in {R}_{+}^{4}$, *there is a stationary distribution μ*(·) *for system* (1.2) *and the ergodicity holds*.

*Proof*. First, we denote

$$b:=3{({d}_{S}+\frac{{\sigma }_{1}^{2}}{2})}^{\frac{2}{3}}[{(\frac{\lambda \beta \theta }{({d}_{E}+\theta +\frac{{\sigma }_{2}^{2}}{2})({d}_{I}+\delta +r+\frac{{\sigma }_{3}^{2}}{2})})}^{\frac{1}{3}}-{({d}_{S}+\frac{{\sigma }_{1}^{2}}{2})}^{\frac{1}{3}}].$$

It is easy to see that *b* > 0. Constructing a *C* ^2^–function $Q:{R}_{+}^{4}\to {R}_{+}$ in the following form

$$\begin{array}{rcl}Q(S,E,I,R) & = & p(-\mathrm{log}\,S-{c}_{1}\,\mathrm{log}\,E-{c}_{2}\,\mathrm{log}\,I)+{(S+E+I+R)}^{\rho +1}\\  &  & -\,\mathrm{log}\,S-\,\mathrm{log}\,E-\,\mathrm{log}\,R\\  & := & p{V}_{1}+{V}_{2}+{V}_{3}+{V}_{4}+{V}_{5},\end{array}$$

where

$${c}_{1}=\frac{{d}_{S}+\frac{{\sigma }_{1}^{2}}{2}}{{d}_{E}+\theta +\frac{{\sigma }_{2}^{2}}{2}},\,{c}_{2}=\frac{{d}_{S}+\frac{{\sigma }_{1}^{2}}{2}}{{d}_{I}+\delta +r+\frac{{\sigma }_{3}^{2}}{2}},$$

*p* and *ρ* are constants satisfying the following condition respectively

22 $$-pb+A+{d}_{S}+\frac{{\sigma }_{1}^{2}}{2}+({d}_{E}+\theta )+\frac{{\sigma }_{2}^{2}}{2}+{d}_{R}+\frac{{\sigma }_{4}^{2}}{2}\le -\mathrm{2,}$$

23 $$f:=({d}_{S}\wedge {d}_{E}\wedge ({d}_{I}+\delta )\wedge {d}_{R})-\frac{1}{2}\rho ({\sigma }_{1}^{2}\vee {\sigma }_{2}^{2}\vee {\sigma }_{3}^{2}\vee {\sigma }_{4}^{2}) > \mathrm{0,}$$

and *A* is determined in the following proof. It is easy to check that

$$\mathop{\mathrm{lim}\,{\rm{\inf }}}\limits_{k\to \infty ,(S,E,I\mathrm{.}R)\in {R}_{+}^{4}\backslash {U}_{k}}\,Q(S,E,I,R)=+\infty ,$$

where ${U}_{k}=(\frac{1}{k},k)\times (\frac{1}{k},k)\times (\frac{1}{k},k)\times (\frac{1}{k},k)$. Besides, *Q*(*S*, *E*, *I*, *R*) is a continuous function. Hence, *Q*(*S*, *E*, *I*, *R*) must have a minimum point (*S*_0_, *E*_0_, *I*_0_, *R*_0_) in the interior of ${R}_{+}^{4}$. Then we define a *C* ^2^– and nonnegative function $V:{R}_{+}^{4}\to {R}_{+}$ as following

$$V(S,E,I,R)=Q(S,E,I,R)-Q({S}_{0},{E}_{0},{I}_{0},{R}_{0}\mathrm{).}$$

According to It*ô*’s formula, we get

$$\begin{array}{rcl}L{V}_{1} & = & -(\frac{\lambda }{S}+\frac{{c}_{1}\beta SI}{\mathrm{(1}+\alpha I)E}+\frac{{c}_{2}\theta E}{I})+\frac{\beta I}{1+\alpha I}+{d}_{S}+{c}_{1}({d}_{E}+\theta )\\  &  & +\,{c}_{2}({d}_{I}+\delta +r)+\frac{{\sigma }_{1}^{2}+{c}_{1}{\sigma }_{2}^{2}+{c}_{2}{\sigma }_{3}^{2}}{2}\mathrm{.}\end{array}$$

Applying inequality $a+b+c\ge 3\sqrt[3]{abc},a,b,c > 0$, yields

24 $$\begin{array}{rcl}L{V}_{1} & \le  & -3{(\frac{{c}_{1}{c}_{2}\lambda \beta \theta }{1+\alpha I})}^{\frac{1}{3}}+\frac{\beta I}{1+\alpha I}+{d}_{S}+{c}_{1}({d}_{E}+\theta )\\  &  & +\,{c}_{2}({d}_{I}+\delta +r)+\frac{{\sigma }_{1}^{2}+{c}_{1}{\sigma }_{2}^{2}+{c}_{2}{\sigma }_{3}^{2}}{2}\\  & = & -3{[\frac{\lambda \beta \theta {({d}_{S}+\frac{{\sigma }_{1}^{2}}{2})}^{2}}{({d}_{E}+\theta +\frac{{\sigma }_{2}^{2}}{2})({d}_{I}+\delta +r+\frac{{\sigma }_{3}^{2}}{2})\mathrm{(1}+\alpha I)}]}^{\frac{1}{3}}\\  &  & +\,3({d}_{S}+\frac{{\sigma }_{1}^{2}}{2})+\frac{\beta I}{1+\alpha I}\\  & = & -3{({d}_{S}+\frac{{\sigma }_{1}^{2}}{2})}^{\frac{2}{3}}[{(\frac{\lambda \beta \theta }{({d}_{E}+\theta +\frac{{\sigma }_{2}^{2}}{2})({d}_{I}+\delta +r+\frac{{\sigma }_{3}^{2}}{2})\mathrm{(1}+\alpha I)})}^{\frac{1}{3}}\\  &  & -{({d}_{S}+\frac{{\sigma }_{1}^{2}}{2})}^{\frac{1}{3}}]+\frac{\beta I}{1+\alpha I}\mathrm{.}\end{array}$$

Similarly,

25 $$\begin{array}{rcl}L{V}_{2} & = & (\rho +\mathrm{1)(}S+E+I+R{)}^{\rho }(\lambda -{d}_{s}S-{d}_{e}E-({d}_{I}+\delta )I-{d}_{R}R)\\  &  & +\frac{1}{2}\rho (\rho +\mathrm{1)(}S+E+I+R{)}^{\rho -1}\\  &  & \times ({\sigma }_{1}^{2}{S}^{2}+{\sigma }_{2}^{2}{E}^{2}+{\sigma }_{3}^{2}{I}^{2}+{\sigma }_{4}^{2}{R}^{2})\\  & \le  & (\rho +\mathrm{1)(}S+E+I+R{)}^{\rho }\\  &  & \times [(\lambda -({d}_{S}\wedge {d}_{E}\wedge ({d}_{I}+\delta )\wedge {d}_{R})(S+E+I+R))]\\  &  & +\frac{1}{2}\rho (\rho +\mathrm{1)(}S+E+I+R{)}^{\rho +1}({\sigma }_{1}^{2}\vee {\sigma }_{2}^{2}\vee {\sigma }_{3}^{2}\vee {\sigma }_{4}^{2})\\  & = & \lambda (\rho +\mathrm{1)(}S+E+I+R{)}^{\rho }\\  &  & -(\rho +\mathrm{1)[(}{d}_{S}\wedge {d}_{E}\wedge ({d}_{I}+\delta )\wedge {d}_{R})\\  &  & -\frac{1}{2}\rho ({\sigma }_{1}^{2}\vee {\sigma }_{2}^{2}\vee {\sigma }_{3}^{2}\vee {\sigma }_{4}^{2})](S+E+I+R{)}^{\rho +1}\\  & \le  & A-\frac{1}{2}(\rho +\mathrm{1)}f{(S+E+I+R)}^{\rho +1}\\  & \le  & A-\frac{1}{2}(\rho +\mathrm{1)}f({S}^{\rho +1}+{E}^{\rho +1}+{I}^{\rho +1}+{R}^{\rho +1}),\end{array}$$

where

$$\begin{array}{rcl}A & = & \mathop{{\rm{\sup }}}\limits_{S+E+I+R\in \mathrm{(0,}\infty )}\{\lambda (\rho +\mathrm{1)(}S+E+I+R{)}^{\rho }\\  &  & -\frac{1}{2}(\rho +\mathrm{1)}f{(S+E+I+R)}^{\rho +1}\} < \infty ,\end{array}\,$$

and *f* is defined in (2.3). We also obtain that

26 $$L{V}_{3}=-\frac{\lambda }{S}+\frac{\beta I}{1+\alpha I}+{d}_{S}+\frac{{\sigma }_{1}^{2}}{2},$$

27 $$L{V}_{4}=-\frac{\beta SI}{\mathrm{(1}+\alpha I)E}+({d}_{E}+\theta )+\frac{{\sigma }_{2}^{2}}{2}$$

and

28 $$L{V}_{5}=-\frac{rI}{R}+{d}_{R}+\frac{{\sigma }_{4}^{2}}{2}.$$

Therefore

$$\begin{array}{ccc}LV & \le  & -3p{({d}_{S}+\frac{{\sigma }_{1}^{2}}{2})}^{\frac{2}{3}}[{(\frac{\lambda \beta \theta }{({d}_{E}+\theta +\frac{{\sigma }_{2}^{2}}{2})({d}_{I}+\delta +r+\frac{{\sigma }_{3}^{2}}{2})(1+\alpha I)})}^{\frac{1}{3}}\\  &  & -{({d}_{S}+\frac{{\sigma }_{1}^{2}}{2})}^{\frac{1}{3}}]+\frac{(p+1)\beta I}{1+\alpha I}\\  &  & -\frac{\beta SI}{(1+\alpha I)E}-\frac{1}{2}(\rho +1)f({S}^{\rho +1}+{E}^{\rho +1}+{I}^{\rho +1}+{R}^{\rho +1})-\frac{\lambda }{S}-\frac{rI}{R}\\  &  & +A+{d}_{S}+\frac{{\sigma }_{1}^{2}}{2}+({d}_{E}+\theta )+\frac{{\sigma }_{2}^{2}}{2}+{d}_{R}+\frac{{\sigma }_{4}^{2}}{2}.\end{array}$$

Consider the following bounded subset

$$D=\{{\epsilon }_{1}\le S\le \frac{1}{{\epsilon }_{1}},{\epsilon }_{1}\le I\le \frac{1}{{\epsilon }_{1}},{\epsilon }_{2}\le E\le \frac{1}{{\epsilon }_{2}},{\epsilon }_{2}\le R\le \frac{1}{{\epsilon }_{2}}\},$$

where ${\epsilon }_{1},{\epsilon }_{2} > 0$ are sufficiently small numbers satisfying the following conditions

29 $$\begin{array}{l}-p\hat{b}+(p+\mathrm{1)}\beta {\epsilon }_{1}+A+{d}_{S}+\frac{{\sigma }_{1}^{2}}{2}+({d}_{E}+\theta )+\frac{{\sigma }_{2}^{2}}{2}+{d}_{R}+\frac{{\sigma }_{4}^{2}}{2}\le -1,\end{array}$$

210 $$-\frac{\lambda }{{\epsilon }_{1}}+A+{d}_{S}+\frac{(p+\mathrm{1)}\beta }{\alpha }+\frac{{\sigma }_{1}^{2}}{2}+({d}_{E}+\theta )+\frac{{\sigma }_{2}^{2}}{2}+{d}_{R}+\frac{{\sigma }_{4}^{2}}{2}\le -1,$$

211 $$\begin{array}{c}-\frac{\beta }{\mathrm{(1}+\alpha {\epsilon }_{1}){\epsilon }_{1}}+3p({d}_{S}+\frac{{\sigma }_{1}^{2}}{2})+\frac{(p+\mathrm{1)}\beta }{\alpha }+A+{d}_{S}+\frac{{\sigma }_{1}^{2}}{2}+({d}_{E}+\theta )\\ \quad +\frac{{\sigma }_{2}^{2}}{2}+{d}_{R}+\frac{{\sigma }_{4}^{2}}{2}\le -1,\end{array}$$

212 $$\begin{array}{c}-\frac{r}{{\epsilon }_{1}^{2}}+3p({d}_{S}+\frac{{\sigma }_{1}^{2}}{2})+\frac{(p+\mathrm{1)}\beta }{\alpha }+A+{d}_{S}+\frac{{\sigma }_{1}^{2}}{2}+({d}_{E}+\theta )\\ \quad +\frac{{\sigma }_{2}^{2}}{2}+{d}_{R}+\frac{{\sigma }_{4}^{2}}{2}\le -1,\end{array}$$

213 $$\begin{array}{c}-\frac{(\rho +\mathrm{1)}f}{2{\epsilon }_{1}^{\rho +1}}+3p({d}_{S}+\frac{{\sigma }_{1}^{2}}{2})+\frac{(p+\mathrm{1)}\beta }{\alpha }+A+{d}_{S}+\frac{{\sigma }_{1}^{2}}{2}+({d}_{E}+\theta )\\ \quad +\frac{{\sigma }_{2}^{2}}{2}+{d}_{R}+\frac{{\sigma }_{4}^{2}}{2}\le -\mathrm{1,}\end{array}$$

214 $$\begin{array}{c}-\frac{(\rho +\mathrm{1)}f}{2{\epsilon }_{2}^{\rho +1}}+3p({d}_{S}+\frac{{\sigma }_{1}^{2}}{2})+\frac{(p+\mathrm{1)}\beta }{\alpha }+A+{d}_{S}+\frac{{\sigma }_{1}^{2}}{2}+({d}_{E}+\theta )\\ \quad +\frac{{\sigma }_{2}^{2}}{2}+{d}_{R}+\frac{{\sigma }_{4}^{2}}{2}\le -1,\end{array}$$

where

$$\begin{array}{lll}\hat{b} & := & -3{({d}_{S}+\frac{{\sigma }_{1}^{2}}{2})}^{\frac{2}{3}}[{(\frac{\lambda \beta \theta }{({d}_{E}+\theta +\frac{{\sigma }_{2}^{2}}{2})({d}_{I}+\delta +r+\frac{{\sigma }_{3}^{2}}{2})\mathrm{(1}+\alpha {\epsilon }_{1})})}^{\frac{1}{3}}\\  &  & -{({d}_{S}+\frac{{\sigma }_{1}^{2}}{2})}^{\frac{1}{3}}].\end{array}$$

We note that for sufficiently small ${\epsilon }_{1}$, condition (2.9) holds due to (2.2). Then

$${R}_{+}^{4}\backslash D={D}_{1}\cup {D}_{2}\cup \cdots \cup {D}_{8},$$

where

$$\begin{array}{rcl}{D}_{1} & = & \{(S,E,I,R)\in {R}_{+}^{4},\,0 < S < {\epsilon }_{1}\},\\ {D}_{2} & = & \{(S,E,I,R)\in {R}_{+}^{4},\,0 < I < {\epsilon }_{1}\},\\ {D}_{3} & = & \{(S,E,I,R)\in {R}_{+}^{4},\,S\ge {\epsilon }_{1},I\ge {\epsilon }_{1},\,0 < E < {\epsilon }_{2}\},\\ {D}_{4} & = & \{(S,E,I,R)\in {R}_{+}^{4},\,I\ge {\epsilon }_{1},\,0 < R < {\varepsilon }_{2}\},\\ {D}_{5} & = & \{(S,E,I,R)\in {R}_{+}^{4},S > \frac{1}{{\varepsilon }_{1}}\},\\ {D}_{6} & = & \{(S,E,I,R)\in {R}_{+}^{4},I > \frac{1}{{\varepsilon }_{1}}\},\\ {D}_{7} & = & \{(S,E,I,R)\in {R}_{+}^{4},E > \frac{1}{{\varepsilon }_{2}}\},\\ {D}_{8} & = & \{(S,E,I,R)\in {R}_{+}^{4},R > \frac{1}{{\varepsilon }_{2}}\}.\end{array}$$

Therefore we consider the following eight cases:

Case 1. If (*S*, *E*, *I*, *R*)∈*D*_1_,

$$\begin{array}{rcl}LV & \le  & -\frac{\lambda }{S}+3p({d}_{S}+\frac{{\sigma }_{1}^{2}}{2})+A+{d}_{S}+\frac{(p+\mathrm{1)}\beta }{\alpha }\\  &  & +\frac{{\sigma }_{1}^{2}}{2}+({d}_{E}+\theta )+\frac{{\sigma }_{2}^{2}}{2}+{d}_{R}+\frac{{\sigma }_{4}^{2}}{2}\\  & \le  & -\frac{\lambda }{{\epsilon }_{1}}+3p({d}_{S}+\frac{{\sigma }_{1}^{2}}{2})+A+{d}_{S}+\frac{(p+\mathrm{1)}\beta }{\alpha }\\  &  & +\frac{{\sigma }_{1}^{2}}{2}+({d}_{E}+\theta )+\frac{{\sigma }_{2}^{2}}{2}+{d}_{R}+\frac{{\sigma }_{4}^{2}}{2}\mathrm{.}\end{array}$$

Then it follows from (2.10) that

$$LV\le -1.$$

Case 2. If (*S*, *E*, *I*, *R*) ∈ *D*_2_, we have

$$\begin{array}{ccc}LV & \le  & -3p{({d}_{S}+\frac{{\sigma }_{1}^{2}}{2})}^{\frac{2}{3}}[{(\frac{\lambda \beta \theta }{({d}_{E}+\theta +\frac{{\sigma }_{2}^{2}}{2})({d}_{I}+\delta +r+\frac{{\sigma }_{3}^{2}}{2})(1+\alpha I)})}^{\frac{1}{3}}-{({d}_{S}+\frac{{\sigma }_{1}^{2}}{2})}^{\frac{1}{3}}]\\  &  & +(p+1)\beta I+A+{d}_{S}+\frac{{\sigma }_{1}^{2}}{2}+({d}_{E}+\theta )+\frac{{\sigma }_{2}^{2}}{2}+{d}_{R}+\frac{{\sigma }_{4}^{2}}{2}\\  & \le  & -p\hat{b}+(p+1)\beta {\varepsilon }_{1}+A+{d}_{S}+\frac{{\sigma }_{1}^{2}}{2}+({d}_{E}+\theta )+\frac{{\sigma }_{2}^{2}}{2}+{d}_{R}+\frac{{\sigma }_{4}^{2}}{2}.\end{array}$$

Using condition (2.9), we obtain

$$LV\le -1.$$

Case 3. If (*S*, *E*, *I*, *R*) ∈ *D*_3_, we obtain that

$$\begin{array}{rcl}LV & \le  & 3p({d}_{S}+\frac{{\sigma }_{1}^{2}}{2})+\frac{(p+\mathrm{1)}\beta }{\alpha }-\frac{\beta SI}{\mathrm{(1}+\alpha I)E}+A\\  &  & +{d}_{S}+\frac{{\sigma }_{1}^{2}}{2}+({d}_{E}+\theta )+\frac{{\sigma }_{2}^{2}}{2}+{d}_{R}+\frac{{\sigma }_{4}^{2}}{2}\\  & \le  & -\frac{\beta {\epsilon }_{1}^{2}}{\mathrm{(1}+\alpha {\varepsilon }_{1}){\varepsilon }_{2}}+3p({d}_{S}+\frac{{\sigma }_{1}^{2}}{2})+A+{d}_{S}\\  &  & +\frac{(p+\mathrm{1)}\beta }{\alpha }+\frac{{\sigma }_{1}^{2}}{2}+({d}_{E}+\theta )+\frac{{\sigma }_{2}^{2}}{2}+{d}_{R}+\frac{{\sigma }_{4}^{2}}{2}.\end{array}$$

Choosing

215 $${\epsilon }_{2}={\epsilon }_{1}^{3},$$

yields

$$\begin{array}{rcl}LV & \le  & -\frac{\beta }{\mathrm{(1}+\alpha {\epsilon }_{1}){\epsilon }_{1}}+3p({d}_{S}+\frac{{\sigma }_{1}^{2}}{2})+A+{d}_{S}+\frac{(p+\mathrm{1)}\beta }{\alpha }\\  &  & +\frac{{\sigma }_{1}^{2}}{2}+({d}_{E}+\theta )+\frac{{\sigma }_{2}^{2}}{2}+{d}_{R}+\frac{{\sigma }_{4}^{2}}{2}\\  & \le  & -1.\end{array}$$

The last inequality holds according to (2.11).

Case 4. If (*S*, *E*, *I*, *R*) ∈ *D*_4_, condition (2.12) combining with (2.15) gives

$$\begin{array}{rcl}LV & \le  & 3p({d}_{S}+\frac{{\sigma }_{1}^{2}}{2})+\frac{(p+\mathrm{1)}\beta }{\alpha }-\frac{rI}{R}+A+{d}_{S}\\  &  & +\frac{{\sigma }_{1}^{2}}{2}+({d}_{E}+\theta )+\frac{{\sigma }_{2}^{2}}{2}+{d}_{R}+\frac{{\sigma }_{4}^{2}}{2}\\  & \le  & -\frac{r{\epsilon }_{1}}{{\epsilon }_{2}}+3p({d}_{S}+\frac{{\sigma }_{1}^{2}}{2})+A+{d}_{S}+\frac{(p+\mathrm{1)}\beta }{\alpha }\\  &  & +\frac{{\sigma }_{1}^{2}}{2}+({d}_{E}+\theta )+\frac{{\sigma }_{2}^{2}}{2}+{d}_{R}+\frac{{\sigma }_{4}^{2}}{2}\\  & \le  & -\frac{r}{{\epsilon }_{1}^{2}}+3p({d}_{S}+\frac{{\sigma }_{1}^{2}}{2})+A+{d}_{S}+\frac{(p+\mathrm{1)}\beta }{\alpha }\\  &  & +\frac{{\sigma }_{1}^{2}}{2}+({d}_{E}+\theta )+\frac{{\sigma }_{2}^{2}}{2}+{d}_{R}+\frac{{\sigma }_{4}^{2}}{2}\\  & \le  & -1.\end{array}$$

Case 5. If (*S*, *E*, *I*, *R*) ∈ *D*_5_, we obtain that

$$\begin{array}{rcl}LV & \le  & 3p({d}_{S}+\frac{{\sigma }_{1}^{2}}{2})+\frac{(p+\mathrm{1)}\beta }{\alpha }-\frac{1}{2}(\rho +\mathrm{1)}f{S}^{\rho +1}+A+{d}_{S}\\  &  & +\frac{{\sigma }_{1}^{2}}{2}+({d}_{E}+\theta )+\frac{{\sigma }_{2}^{2}}{2}+{d}_{R}+\frac{{\sigma }_{4}^{2}}{2}\\  & \le  & -\frac{(\rho +\mathrm{1)}f}{2{\epsilon }_{1}^{\rho +1}}+3p({d}_{S}+\frac{{\sigma }_{1}^{2}}{2})+\frac{(p+\mathrm{1)}\beta }{\alpha }+A\\  &  & +{d}_{S}+\frac{{\sigma }_{1}^{2}}{2}+({d}_{E}+\theta )+\frac{{\sigma }_{2}^{2}}{2}+{d}_{R}+\frac{{\sigma }_{4}^{2}}{2},\end{array}$$

this together with (2.13), we derive that

$$LV\le -1.$$

Case 6. If (*S*, *E*, *I*, *R*) ∈ *D*_6_,

$$\begin{array}{rcl}LV & \le  & 3p({d}_{S}+\frac{{\sigma }_{1}^{2}}{2})+\frac{(p+\mathrm{1)}\beta }{\alpha }-\frac{1}{2}(\rho +\mathrm{1)}f{I}^{\rho +1}+A+{d}_{S}\\  &  & +\frac{{\sigma }_{1}^{2}}{2}+({d}_{E}+\theta )+\frac{{\sigma }_{2}^{2}}{2}+{d}_{R}+\frac{{\sigma }_{4}^{2}}{2}\\  & \le  & -\frac{(\rho +\mathrm{1)}f}{2{\epsilon }_{1}^{\rho +1}}+3p({d}_{S}+\frac{{\sigma }_{1}^{2}}{2})+\frac{(p+\mathrm{1)}\beta }{\alpha }+A+{d}_{S}\\  &  & +\frac{{\sigma }_{1}^{2}}{2}+({d}_{E}+\theta )+\frac{{\sigma }_{2}^{2}}{2}+{d}_{R}+\frac{{\sigma }_{4}^{2}}{2}\\  & \le  & -\mathrm{1,}\end{array}$$

which follows from (2.13).

Case 7. If (*S*, *E*, *I*, *R*) ∈ *D*_7_, it follows that

$$\begin{array}{rcl}LV & \le  & 3p({d}_{S}+\frac{{\sigma }_{1}^{2}}{2})+\frac{(p+\mathrm{1)}\beta }{\alpha }-\frac{1}{2}(\rho +\mathrm{1)}f{E}^{\rho +1}+A\\  &  & +{d}_{S}+\frac{{\sigma }_{1}^{2}}{2}+({d}_{E}+\theta )+\frac{{\sigma }_{2}^{2}}{2}+{d}_{R}+\frac{{\sigma }_{4}^{2}}{2}\\  & \le  & -\frac{(\rho +\mathrm{1)}f}{2{\epsilon }_{2}^{\rho +1}}+3p({d}_{S}+\frac{{\sigma }_{1}^{2}}{2})+\frac{(p+\mathrm{1)}\beta }{\alpha }+A+{d}_{S}\\  &  & +\frac{{\sigma }_{1}^{2}}{2}+({d}_{E}+\theta )+\frac{{\sigma }_{2}^{2}}{2}+{d}_{R}+\frac{{\sigma }_{4}^{2}}{2},\end{array}$$

which together with (2.14) implies that

$$LV\le -1.$$

Case 8. If (*S*, *E*, *I*, *R*) ∈ *D*_8_, we obtain that

$$\begin{array}{rcl}LV & \le  & 3p({d}_{S}+\frac{{\sigma }_{1}^{2}}{2})+\frac{(p+\mathrm{1)}\beta }{\alpha }-\frac{1}{2}(\rho +\mathrm{1)}f{R}^{\rho +1}+A+{d}_{S}\\  &  & +\frac{{\sigma }_{1}^{2}}{2}+({d}_{E}+\theta )+\frac{{\sigma }_{2}^{2}}{2}+{d}_{R}+\frac{{\sigma }_{4}^{2}}{2}\\  & \le  & -\frac{(\rho +\mathrm{1)}f}{2{\epsilon }_{2}^{\rho +1}}+3p({d}_{S}+\frac{{\sigma }_{1}^{2}}{2})+\frac{(p+\mathrm{1)}\beta }{\alpha }+A+{d}_{S}\\  &  & +\frac{{\sigma }_{1}^{2}}{2}+({d}_{E}+\theta )+\frac{{\sigma }_{2}^{2}}{2}+{d}_{R}+\frac{{\sigma }_{4}^{2}}{2}\mathrm{.}\end{array}$$

Combining (2.14), yields

$$LV\le -1.$$

Based on the discussion of the above eight cases, we obtain

216 $$LV\le -1,\,(S,E,I,R)\in {R}_{+}^{4}\backslash D.$$

Therefore, *A*_2_ in Lemma 2.1 is satisfied. In addition, *A*_1_ is also satisfied (see ref. 17). Theorem 2.2 is proved according to Lemma 2.1.☐

**Remark 2.1**. Yang *et al*.^17^ have studied system (1.2), in Theorem 3.3 they show that under conditions (H1) and (H2), there is a stationary distribution *μ*(·) for the system. Comparing with Theorem 2.2 in our investigation, we only need condition ${R}_{0}^{s} > 1$, without other conditions imposed on the coefficients. That is to say, Theorem 2.2 in large improves Theorem 3.3 in ref. 17. Moreover, we see that if *σ*_*i*_ = 0 (*i* = 1, 2, 3, 4), the above condition is reduced to *R*_0_ > 1, which is the condition for globally asymptotically stable of endemic equilibrium P* of system (1.1). And ${R}_{0}^{s}$ is smaller than the basic reproduction number *R*_0_ of system (1.1).
